# Toward Targeted Therapies in Oesophageal Cancers: An Overview

**DOI:** 10.3390/cancers14061522

**Published:** 2022-03-16

**Authors:** Giacomo Bregni, Benjamin Beck

**Affiliations:** 1 Institut Jules Bordet, Université Libre de Bruxelles (ULB), 1050 Brussels, Belgium; giacomo.bregni@bordet.be; 2 Welbio and FNRS Investigator at IRIBHM, Faculty of Medicine, Université Libre de Bruxelles (ULB), 1050 Brussels, Belgium

**Keywords:** oesophagus, cancer, therapeutics

## Abstract

**Simple Summary:**

Prognosis for patients with oesophageal cancer is poor, because of its aggressive nature and the lack of targeted therapies. Advances in cancer biology and sequencing technology have enabled the selection of targeted therapies for individual patients with various types of tumors, such as breast or lung cancers as well as melanoma. However, precision oncology for patients with oesophageal cancer is still virtually non-existent. This review outlines the recent advances in oesophageal molecular profiling and the outcome of clinical trials based on targeted therapies in this disease. The signaling pathways that should be further investigated and the impact of tumor heterogeneity on resistance to therapy are also discussed.

**Abstract:**

Oesophageal cancer is one of the leading causes of cancer-related death worldwide. Oesophageal cancer occurs as squamous cell carcinoma (ESCC) or adenocarcinoma (EAC). Prognosis for patients with either ESCC or EAC is poor, with less than 20% of patients surviving more than 5 years after diagnosis. A major progress has been made in the development of biomarker-driven targeted therapies against breast and lung cancers, as well as melanoma. However, precision oncology for patients with oesophageal cancer is still virtually non-existent. In this review, we outline the recent advances in oesophageal cancer profiling and clinical trials based on targeted therapies in this disease.

## 1. Introduction

Oesophageal cancer is one of the leading causes of cancer-related death across the world. With about 600,000 cases per year worldwide, it is the seventh most common and sixth most lethal cancer, determining 1 out of 18 cancer-related deaths in 2020 [1]. Approximately 19,000 new cases and 15,500 deaths were expected in the United States of America (US) in 2021 [2], unevenly divided between males and females. The incidence of oesophageal cancer varies widely based on geographic regions [3]: oesophageal cancer occurs more frequently in developing countries, with a disproportionate number of cases arising in China, while adenocarcinoma is more evenly distributed across the world [4]. Differences in incidence between men and women are pronounced in low-incidence countries and not as evident in high-incidence countries, likely due to the differences in underlying risk factors [5].

The geographic difference largely reflects the divide between the two most common oesophageal cancer histologies, squamous cell carcinoma (ESCC) and adenocarcinoma (EAC) [6]. ESCC prevalence is concentrated in three main regions, central-eastern Asia, the eastern coast of Africa, and an area in south America centered around Uruguay, which carry an incidence up to 10-fold higher than non-endemic areas [5]. Significant variations also occur inside high-incidence areas. While China as a whole is home to approximately half of all oesophageal cancers worldwide, the distribution varies widely, with some small areas having ESCC as one of the leading causes of death overall [7]. Even outside of high-incidence areas, ESCC is the most frequent histology in developing countries, and associated risk factors include cigarette smoking, alcohol consumption, and dietary characteristics [8]. Elevated consumption of red meat, salted meat, and products inducing thermal injury, such as mate, hot teas, and soups, has been shown to increase the risk of ESCC in high-incidence countries [8,9]. Additional risk factors include the contamination of food with carcinogenic compounds, namely, a high concentration of nitrosamine in the diet of populations in areas of China [4,10]. Interestingly, smoking seems to play a small role as a risk factor in countries with a high incidence of ESCC, probably due to the higher relative relevance of exposition to polycyclic aromatic hydrocarbons from dietary and/or environmental sources [11,12]. Conversely, the reduction in smoking rates in recent years seems to have significantly impacted the incidence of ESCC in Western countries, particularly among males [13,14]. In addition to environmental risk factors, some germline variants have been shown to increase the risk of development of ESCC. Notably, variants in the Breast Cancer 2 (*BRCA2*) gene in the Iran Turkmen and in the Chinese populations suggest a role for defective homologous recombination in ESCC carcinogenesis [15,16]. This suspicion is reinforced by the association between pathogenic variants in the Fanconi Anemia Complementation Group D2 (*FANCD2*) gene and the risk of ESCC [17]. *FANCD2* is a gene involved in cell cycle regulation and in Fanconi Anemia, a genomic instability disorder [18]. Fanconi Anemia patients with pathogenic variants in the *FANCD2* are at high risk of developing ESCC, probably due to the role of this gene in accelerating cell cycle progression [19].

Meanwhile, the incidence of EAC has been increasing during the last 30 years, particularly in Western countries, where it is now the most frequent oesophageal cancer histology [20]. The incidence of EAC increases with age, and it shows a striking difference between males and females, with a male to female ratio as high as 9:1 in the USA [21]. The difference in distribution between EAC and ESCC is likely related to the divergent risk factors associated with each histology. Multiple risk factors associated with lifestyle have been linked with EAC, including gastroesophageal reflux, Barrett′s oesophagus, alcohol consumption, tobacco smoking, obesity, sedentary lifestyle, and lack of physical activity [20]. The interlink among some of these lifestyle-related risk factors might explain part of the familial clustering of Barrett′s oesophagus and EAC [22]: patients with a first-degree relative with Barrett′s oesophagus or EAC are at increased risk of developing Barrett′s oesophagus, likely due to a combination of environmental and genetic risk factors [23]. Several germline variants have in fact been associated with the development of Barrett′s oesophagus and/or EAC, and up to one third of EAC may have a hereditary component [24,25]. Nevertheless, the increased risk potentially associated with each mutation is limited, and no mutation has demonstrated to increase the risk by more than 20%.

## 2. Prognosis and Current Treatment Advancements

Prognosis for patients with either ESCC or EAC is poor, with less than 20% of patients surviving more than 5 years after diagnosis [26]. The prognosis of oesophageal cancer patients can be stratified based on the presence (stage IV) or absence (stages I-III, localized or locally advanced disease) of distant metastases. Considering the two histologies combined, 5-year survival rates range from 46.4% for localized tumors to 5.2% for stage IV in the US [27]. Survival for stage IV patients does not differ significantly between ESCC and EAC, with 5-year overall survival (OS) ranging from 5.0 to 7.5% and 4.3 to 5.8%, respectively. In the past 15 years, the survival for stage IV patients with oesophageal cancer has slightly improved, despite no significant advancements in the standard of care [3].

The similar lack of advancements in terms of survival between the two histologies reflects to the uniformity in the treatment of these patients. The Erb-B2 Receptor Tyrosine Kinase 2 (*ERBB2*) gene encodes for the human epidermal growth factor 2 (HER2) protein involved in cell growth [28], and it is frequently overexpressed/amplified in several tumor types [29]. Inhibitors of HER2 have shown efficacy in breast, gastric, and colorectal cancer, becoming part of the standard of care in patients affected by these tumors [30,31,32]. Aside from EAC patients with overexpression/amplification of *HER2*, who are frequently treated according to treatment protocols first established for gastric cancers [33], no significant differences currently exist regarding the systemic treatment of EAC or ESCC, and clinical trials specifically dedicated to either EAC or ESCC are infrequent. After several years with no significant improvement in the standard of care, the first-line treatment of unresectable and metastatic oesophageal cancer has recently been changed by the addition of immunotherapy, alone or in combination with a chemotherapy backbone (Table 1) [34]. The phase III randomized KEYNOTE-590 tested the addition of pembrolizumab to cisplatin and 5-fluorouracil in the first-line treatment of EAC, ESCC, and gastro-oesophageal junction (GEJ) patients irrespective of programmed death-ligand 1 (PD-L1) status [35]. PD-L1 is a transmembrane protein involved in immune suppression, and the interaction between PD-L1 and programmed death protein 1 (PD1) is the target of most of the currently available immune checkpoint inhibitors [36]. The expression of PD-L1 is frequently assessed in several tumor types because of its association with benefit from immunotherapy, and it can be evaluated according to several different scores [37]. One of the most frequently used is the combined positive score (CPS), which takes into account the expression of PD-L1 on tumor cells, lymphocytes, and macrophages combined [38]. Among the 749 patients enrolled in the KEYNOTE-590 study, the combination of pembrolizumab plus chemotherapy proved superior to placebo plus chemotherapy in terms of OS and progression-free survival (PFS) in all randomized patients (OS: hazard ratio (HR) 0.73 [95%CI 0.62–0.86], *p* < 0.0001; PFS: HR 0.65 [95%CI 0.55–0.760]; *p* < 0.0001), and in patients with EAC, ESCC, or GEJ and CPS equal or superior to 10 (OS: HR 0.62 [95%CI 0.49–0.78]; *p* < 0.0001; PFS: HR 0.51 [95%CI 0.41–0.65]; *p* < 0.0001). The results of this study led to the approval by European regulatory authorities “*for the first-line treatment of patients with locally advanced unresectable or metastatic carcinoma of the oesophagus or HER-2 negative gastroesophageal junction adenocarcinoma in adults whose tumours express PD-L1 with a CPS ≥ 10*” [39]. Checkmate-649 tested a similar strategy, comparing a chemotherapy plus nivolumab arm, a nivolumab plus ipilimumab arm, and a chemotherapy-only arm in patients with advanced oesophageal, GEJ, and gastric cancer [40]. The study aimed at demonstrating the superiority in terms of OS and PFS of chemotherapy plus nivolumab versus chemotherapy alone in the population with CPS equal or superior to 5. In the 955 enrolled patients with a CPS equal or superior to 5, the chemotherapy plus immunotherapy arm proved to be superior to chemotherapy only, with a median OS of 14.1 versus 11.1 months (HR 0.71 [98.4%CI 0.59–0.86]; *p* < 0.0001) and a median PFS of 7.7 versus 6.0 months (HR 0.68 [98%CI 0.56–0.81]; *p* < 0.0001). Of note, oesophageal cancer patients constituted only 12% of the enrolled population. Among the few studies enrolling only a specific subtype of oesophageal cancer, CheckMate-648 was a 3-arm phase III study, randomizing patients to cisplatin plus 5-fluorouracil plus nivolumab, cisplatin plus 5-fluorouracil, or nivolumab plus ipilimumab [41]. The study enrolled patients with ESCC irrespective of PD-L1 status, but the co-primary endpoints were assessed in patients with tumor cell PD-L1 equal or superior to 1%. Of the 970 total patients, 49% had PD-L1 equal or more than 1%. The chemotherapy plus immunotherapy arm showed a significant superiority in terms of OS (HR 0.54, [99.5%CI 0.37–0.80]; *p* < 0.0001) and PFS (HR 0.65 [98.5%CI 0.46–0.92]; *p* = 0.0023) versus chemotherapy alone, while the immunotherapy-only arm only proved to be superior in terms of OS (HR 0.64 [98.6%CI 0.46–0.90]; *p* = 0.001), with the advantage in PFS not reaching the statistical significance boundary.

Immune checkpoint inhibitors have also entered later lines of treatment in oesophageal cancer, mainly restricted to the squamous cell histology. The KEYNOTE-181 study demonstrated the superiority of pembrolizumab versus the investigator′s choice of standard chemotherapy treatments (paclitaxel, docetaxel, or irinotecan) in the second line treatment of patients with ESCC, EAC, or GEJ cancer with CPS ≥ 10 [42]. Out of the 628 total patients enrolled in the clinical trial, 35% had CPS ≥ 10. In this population, median OS was significantly longer in patients receiving pembrolizumab as opposed to standard chemotherapy (HR 0.69 [95%CI 0.52–0.93]; *p* = 0.0074). In a subgroup analysis of the CPS ≥ 10 population, the advantage remained significant only for ESCC patients, leading to the Food and Drug Administration (FDA) approval of pembrolizumab for ESCC patients with CPS ≥ 10 progressing after one or more prior lines of systemic treatment [39]. Nivolumab too proved effective after the first line of treatment in ESCC, showing superiority over paclitaxel or docetaxel in terms of overall survival (HR 0.77 [95%CI 0.62–0.96]; *p* = 0.019) in previously treated patients enrolled in the ATTRACTION-3 study [43]. Thanks to the results of this trial, nivolumab is currently FDA approved for ESCC patients who have previously received a platinum and fluoropyrimidine-based treatment [44].

It is also worth mentioning, although the treatment of non-metastatic patients goes beyond the scope of this review, the relevant impact that the introduction of immune checkpoint inhibitors has had on the adjuvant setting in oesophageal cancer. The CheckMate 577 trial showed that in oesophageal or GEJ cancer patients with residual disease after neoadjuvant chemoradiotherapy and surgery, post-operative nivolumab doubles median disease-free survival compared to placebo (22.4 versus 11.0 months, HR 0.69 [96.4%CI 0.56–0.86]; *p* < 0.001) [45]. Although the overall survival data have not been presented yet, the results of this trial have already changed clinical practice in patients treated with neoadjuvant chemoradiotherapy followed by surgery [34].

Following decades marked by limited improvement in terms of overall survival, the beneficial effect of immune checkpoint inhibitors in localized, locally advanced, and metastatic oesophageal cancer patients is likely to become apparent in the next few years.

## 3. Molecular Characterization of Oesophageal Cancers

There is growing interest in the development of combinations of immunotherapy and targeted therapies [46,47] Indeed, studies in animal models and clinical studies showed drug-dependent and dose-dependent interactions between chemotherapy and the immune system that could be used to induce synergy between cytotoxic drugs and immunotherapy in several cancer types [48] In spite of the beneficial effect of immune checkpoint inhibitors in oesophageal cancer, there is thus still a need to develop novel targeted therapies. This requires a profound characterization of esophageal cancers at the molecular level and a better understanding of their heterogeneity.

### 3.1. Heterogenity of Oesophageal Squamous Cell Carcinoma

Despite similarities in terms of treatment and prognosis, ESCC and EAC bear clearly distinct molecular profiles. Gene expression analysis shows upregulation of Wnt, syndecan and p63 pathways in ESCC, while EAC is characterized by higher E-cadherin signaling, together with higher expression of pathways regulating E-cadherin, such as ARF6 and FOXA pathways [44]. The two histologies also differ in terms of somatic genomic mutations and somatic copy-number alterations. Although most of the genomic alterations in oesophageal cancers do not currently have a clinical utility, understanding the molecular profiles of oesophageal cancers can help foster the development of future targeted therapies. In series exploring the clinical utility of genomic sequencing in gastro-oesophageal cancers as a whole, 35–43% of tumors have been shown to harbor at least one alteration considered “actionable” (i.e., potentially responsive to a targeted therapy), suggesting that the deepening of our knowledge of the molecular landscape of oesophageal cancer might open new therapeutic possibilities for these patients [49,50,51].

**Figure 1:**
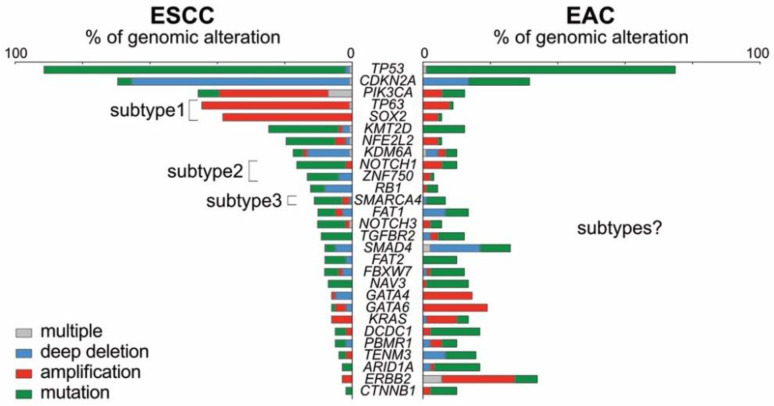
Barplot summarizing frequent genomic alterations found in ESCC and EAC (only alterations >10% of samples in at least one histology are represented). These data highlight differences in the pattern of genomic alterations between ESCC and EAC. Three ESCC molecular subtypes are defined based on the genomic alteration profile. Data from the TCGA, Firehose Legacy (*n* = 186). Abbreviations: EAC: oesophageal adenocarcinoma; ESCC: oesophageal squamous cell carcinoma; TP53: Tumor Protein P53; CDKN2A: Cyclin Dependent Kinase Inhibitor 2A; PIK3CA: Phosphatidylinositol-4,5-Bisphosphate 3-Kinase Catalytic Subunit Alpha; TP63: Tumor Protein P63; SOX: Sry-type HMG box; KMT2D: Lysine Methyltransferase 2D; NFE2L2: Nuclear factor erythroid 2-related factor 2; KDM6A: Lysine Demethylase 6A; NOTCH1: Notch homolog 1, translocation-associated; ZNF750: Zinc Finger Protein 750; RB1: Retinoblastoma; SMARCA4: SWI/SNF-Related, Matrix-Associated, Actin-Dependent Regulator Of Chromatin, Subfamily A, Member 4; FAT1: FAT Atypical Cadherin 1; NOTCH3: Notch Receptor 3; TGFBR2: Transforming Growth Factor Beta Receptor 2; SMAD4: SMAD family member 4; FAT2: FAT Atypical Cadherin 2; FBXW7: F-Box And WD Repeat Domain Containing 7; NAV3: Neuron Navigator 3; GATA4: GATA Binding Protein 4; GATA6: GATA Binding Protein 6; KRAS: Kirsten rat sarcoma virus; DCDC1: Doublecortin Domain Containing 1; PBMR1: Polybromo 1; TENMR3: Teneurin Transmembrane Protein 3; ARID1A: AT-Rich Interaction Domain 1A; *ERBB2*: Erb-B2 Receptor Tyrosine Kinase 2; CTNNB1: Catenin Beta 1.

Frequently mutated genes in ESCC include TP53, NOTCH1, NFE2L2, CDKN2A, PIK3CA, RB1, FAM135, ADAM29, MLL2, FBXW7, AJUBA, CREEBP, PTCH1, ZNF750, PTEN, FAT1, EP300, FAT2, KDM6A, CREBBP, BAP1, NOTCH3, TGFBR2, CUL3, DCDC1, NAV3, TENM3, TET2, RIPK4, PBRM1 and USP8 [44,52,53,54,55,56,57,58] (Figure 1, including full gene names).

In addition, several mutational signatures associated have been found in ESCC, some associated with known risk factors [59,60]. Tobacco smoking has been shown to be associated to the COSMIC database signature single base substitutions 4 (SBS4) (C > A), alcohol consumption with signatures SBS16 (T > C) and ID11, opium exposure to SBS288J_Iran (T > C), and deamination of 5-methylcytosine to SBS1 (C > T). Six mutational signatures (SBS1, SBS2, SBS5, SBS13, SBS18 and SBS40) account for more than 80% of the mutational burden of ESCC, with apolipoprotein B mRNA editing enzyme, and catalytic polypeptide-like (APOBEC)-associated signatures being present in approximately 90% of cases, supporting the contention that APOBEC alterations are fundamental in the development of ESCC [60].

At least three subtype classifications based on molecular characteristics have been developed for ESCC [44,61,62]. The TCGA (The Cancer Genome Atlas Program) classification divided ESCC tumors into three molecular subtypes: ESCC1, ESCC2 and ESCC3 (Figure 1). ESCC1 shows a high frequency of alterations in the Nuclear Respiratory Factor 2 (NRF2) pathway, which plays a role in response to oxidative stress. Mutations in *NFE2L2*, Cullin 3 (*CUL3)*, and Kelch-Like ECH-Associated Protein 1 (*KEAP1)* are common in this subtype, as well as the amplification of *SOX2* and/or *TP63*. ESCC1 samples closely resemble squamous cell carcinoma of lung and head and neck origin [44]. ESCC2 is characterized by the high frequency of mutations in the epithelial-to-mesenchymal transition (EMT)-associated gene *NOTCH1* and epidermal cell differentiator *ZNF750* [63,64], and by greater leukocyte infiltration as compared to the other two subtypes. In ESCC3, all samples had alterations activating the PIK3 pathway, while most of them had no cell cycle dysregulation. Among squamous cell cancers, ESCC3 molecular characteristics seem to be exclusive to ESCC. Given the incidence of ESCC in China, Liu and coauthors proposed a further molecular classification specific to Chinese patients [62]. Subtype 1 is characterized by the upregulation of pathways involved in the regulation of cell metabolism, Subtype 2 by inflammation, immune cell, and cytokine signaling, and Subtype 3 by the upregulation of genes associated with cell cycle and cell proliferation. A third molecular subtyping classification of ESCC samples based on proteomics profile proposed two subtypes with potential clinical implications [61]. Tumors in the S2 subtype show an extreme expression pattern, with dysregulated proteins either severely downregulated or upregulated. The expression pattern of this subtype, enriched in DNA replication, DNA repair, and G2/M checkpoint pathways, suggests a progressive evolution from non-tumor to S1 tumor to S2 tumor samples. Tumors in the S2 subtype also show a significantly worse prognosis compared to S1.

While harmonization efforts among different molecular subtypes have been undertaken in other tumor types, such as the consensus molecular subtypes in colorectal cancer [65], to the best of our knowledge no such study exists in ESCC. Studies aiming at the standardization of existing molecular classifications would build towards the use of these tools for the molecular selection of patients for clinical trials.

In addition to intertumor variations, intratumor heterogeneity (i.e., the presence of different molecular clones inside a single tumor; ITH) must also be taken into account when dealing with the molecular characterization of ESCC. In an ESCC cohort subjected to whole exome sequencing, 35.8% of somatic mutations and 90% of recurrent copy number alterations were found to be spatially heterogeneous [66]. Given the huge impact of ITH on drug resistance, this degree of heterogeneity might partly explain the dismal prognosis associated with ESCC [67].

### 3.2. Heterogeneity of Oesophageal Adenocarcinoma

Driver mutations associated with the development of EAC include *TP53*, *CDKN2A*, *SMAD4*, *ARID1A*, *ERBB2*, *KRAS*, *PIK3CA*, *SMARCA4*, *CTNNB1*, *ARID2*, *PBRM1* and *FBXW7* [44,68,69].

The molecular characteristics of EAC closely resemble those of gastric cancers, particularly of the CIN molecular subtype, with almost all the EAC tumors assessed in the TCGA cohort being classified in the chromosomal instability (CIN) subtype of gastric cancer [44]. A molecular classification specific to EAC categorized tumors into three subtypes based on their genomic landscape [69]. Whole genome sequencing of a large cohort of samples showed that EAC is characterized by copy number changes and by large-scale genomic events, with a high frequency of genomic catastrophes such as chromothripsis, kataegis, and complex rearrangements. Three subtypes were described: C > A/T dominant, with C > A/T mutational pattern and age as risk factor, DNA damage response (DDR) impaired, identified by homologous recombination defects, and mutagenic, the subtype with the highest mutational burden. Two further classifications based on DNA methylation profiles have recently been proposed [70,71]. Methylation aberrations are present in both EAC and Barrett′s oesophagues (BE), and they are suspected to be involved in the progression from BE to EAC.

Somatic copy-number alterations (SCNA) analysis also demonstrates similarities and differences between ESCC and EAC, with *VEGFA*, *ERBB2*, *GATA6* and *CCNE1* amplified in EAC but not in ESCC, and *SOX2*, *TERT*, *FGFR1*, *MDM2*, and *NKX2-1* amplified in ESCC but not in EAC [44]. Regarding deletions, *SMAD4* and *RB1* are frequently deleted in EAC and ESCC, respectively [44].

The analysis of oesophageal carcinomas using integrated clustering of SCNA, DNA methylation, mRNA and microRNA expression data by the TCGA call into question the premise of envisioning oesophageal cancer as a single entity. Indeed, these data clearly show that histological subtypes of EAC and ESCC are distinct in their molecular characteristics and should therefore be treated as distinct malignancies.

Similar to ESCC, intratumor heterogeneity is a significant issue also in EAC. An analysis performed on a limited number of EACs showed the presence of spatial heterogeneity in each sample of the cohort, with a median of more than 50% of nonsilent mutations being heterogeneous [72]. Moreover, tumors with high ITH were shown to be less sensitive to neoadjuvant platinum-based chemotherapy [72]. This might have significant implications for the use of targeted treatments in EAC patients.

## 4. Targeted Treatments in Oesophageal Cancer: Not There Yet

Despite abundant data on molecular alterations in oesophageal cancer, targeted treatments are virtually non-existent in the therapeutic landscape of these tumors, if we exclude the use of the anti-VEGF ramucirumab and of the anti-HER2 trastuzumab in GEJ cancers [32,73,74]. Patients with EAC showing overexpression or amplification of ERBB2 commonly receive a first-line treatment consisting of chemotherapy plus the anti-HER2/neu monoclonal antibody trastuzumab, according to the therapy regimen tested in a phase III trial enrolling GEJ and gastric cancer patients [33]. Metastatic oesophageal cancer, particularly the adenocarcinoma histology, is frequently treated following the recommendations for gastric cancer, despite limited evidence of the associated benefit [75]. Beside this, most of the targeted therapies tested for the past 10 years are aimed at Receptor Tyrosine Kinases (RTKs) such as EGFRs and VEGFRs.

### 4.1. Targeting the EGFR Pathway in Oesophageal Cancers

Epidermal growth factor receptor (*EGFR*) amplification is a frequent alteration in both ESCC and EAC, with a frequency ranging between 7 and 28% in different cohorts [76]. Of the phase III trials testing targeted treatments conducted in patients with oesophageal cancer in recent years, most aimed at targeting EGFR (Table 2) [77,78,79]. The REAL3 trial tested the addition of the anti-EGFR monoclonal antibody panitumumab to a chemotherapy regimen of epirubicin, oxaliplatin, and capecitabine in patients with oesophageal, GEJ, or gastric cancer [78]. Out of the 553 assessable subjects, 217 (39%) had oesophageal cancer. Median OS resulted to be superior in patients treated in the standard arm in the overall population (8.8 versus 11.3 months; HR 1.37 [95%CI 1.07–1.76]; *p* = 0.013) as well as in the prespecified oesophageal cancer subpopulation (HR 1.32 [95%CI 0.90–1.94]). No significant difference between treatment groups was observed in terms of PFS (1.22 [95%CI 0.98–1.52]; *p* = 0.068). A correlative analysis of this study aimed at assessing the association between EGFR amplification and survival outcomes in patients with available samples in the intention-to treat (ITT) population [80]. *EGFR* amplification (copy number variation score ≥ 2 or ≥5) associated with worse PFS and OS both in the study population and in an extended in silico analysis performed in the cBioportal database. Moreover, panitumumab had no added benefit in the EGFR-amplified population, and data from patient-derived organoids showed a potential detrimental antagonistic effect between anti-EGFR agents and epirubicin dependent on an accelerated M-to-G1 transition in EGFR amplified tumors. The COG trial enrolled patients with EAC, ESCC, or GEJ cancer who had received up to previous lines of treatment for unresectable or metastatic disease [77]. Patients were randomized to receive the EGFR inhibitor gefitinib or placebo, and the primary endpoint of the study was overall survival. Gefitinib failed to demonstrate a survival benefit compared to placebo, with a median OS of 3.73 and 3.67 months, respectively (HR 0.90 [95%CI 0.74–1.09]; *p* = 0.293). PFS resulted to be slightly longer in the gefinitib than in the placebo arm (HR 0.80 [95%CI 0.66–0.96]; *p* = 0.020). In the 76% of enrolled patients who had tissue available for molecular analyses, EGFR fluorescence in situ hybridization (FISH)-positive tumors derived a survival benefit in terms of OS and PFS from gefitinib, while *KRAS*, *PIK3CA*, and *BRAF* mutations did not influence survival outcomes [81]. Finally, the AIO/EORTC POWER trial randomized a total of 146 ESCC patients to receive either cisplatin plus 5-fluorouracil or the same chemotherapy regimen plus panitumumab [79]. Once again, the addition of panitumumab did not result in a survival benefit, and the study was stopped early for a higher mortality rate in the experimental arm (HR 1.77 [95%CI 1.06–2.98]; *p* = 0.028). The expression of EGFR, MET, and CXCR4 in tumor tissue samples had no association with the survival outcomes. A further phase III trial enrolling a small population with oesophageal adenocarcinoma (20 patients out of a total efficacy population of 487) was the TRIO-013/LOGIC trial, which randomized subjects into two arms: CAPOX plus placebo versus CAPOX plus the anti-HER2 agent lapatinib [82]. In the primary efficacy population, composed of patients with tumors showing HER2 amplification, lapatinib led to no improvement in terms of overall survival (HR 0.91 [95%CI 0.73–1.12]; *p* = 0.349).

Combinations of agents targeting the mitogen-activated protein kinase (MAPK) pathway and chemotherapy or immunotherapy have also been explored in several phase II trials. Erlotinib, cetuximab, and panitumumab have all been studied in combination with chemotherapy regimens in oesophageal cancer. Panitumumab plus irinotecan, panitumumab plus DCF (docetaxel plus cisplatin plus fluorouracil), and erlotinib plus FOLFOX all failed to demonstrate significant activity in this disease [83,84,85]. Based on their potential synergistic effect, a single-arm phase II study administered a combination of the anti-HER2 agent trastuzumab, platinum-based doublet chemotherapy, and the immune checkpoint inhibitor pembrolizumab to 37 patients with HER2 overexpressed or amplified oesophageal, GEJ, or gastric cancer [86]. The combination led to promising results in terms of PFS (median PFS 13.0 months) and overall survival (median OS 27.3 months), prompting the design of a currently enrolling phase III trial in patients with GEJ or gastric cancer [87].

Several phase II studies have tested single agents targeting the EGFR pathway. The pan-ErbB kinase inhibitor afatinib was recently tested in a single-arm phase II study enrolling patients refractory to platinum-based chemotherapy [88]. Afatinib showed modest clinical activity, with an overall response rate of 14.3%. Other anti-EGFR agents that have been explored as monotherapies for advanced or metastatic oesophageal cancer in phase II studies include icotinib, dacomitinib, erlotinib, and cetuximab [89,90,91,92]. In populations composed of ESCC pre-treated patients selected for EGFR overexpression or amplification, the oral tyrosine kinase inhibitor (TKI) icotinib led to an ORR of 16.7% [92] while, in a similar population unselected for EGFR status, the pan-HER in-hibitor dacomitinib showed an ORR of 12.5% [89,90,91,92]. Erlotinib and cetuximab both showed limited activity in oesophageal cancer [89,90,91].

In patients with inoperable oesophageal cancer, a common research strategy has been the combination of radiation therapy and anti-EGFR agents. The oral tyrosine kinase inhibitor icotinib was recently investigated together with radiation therapy in a randomized phase II study enrolling patients aged 70 years or older [93]. The combination of icotinib and radiotherapy determined a significative improvement in terms of the primary endpoint overall survival compared to radiation therapy alone (median OS 24.0 versus 16.3, HR 0.53 [95%CI 0.33–0.87]; *p* = 0.008). Several trials have tested similar strategies employing a combination of agents targeting members of EGFR family (mainly HER2 and EGFR) and radiation therapy, with mixed results [94,95,96,97,98,99,100,101,102,103,104,105,106,107].

The disappointing results of anti-EGFR treatments in oesophageal cancer somehow reflect what has been observed in gastric cancer, in which no clinical trial has been able to demonstrate a survival advantage so far [108,109]. On the contrary, the anti-EGFR antibody cetuximab is a standard treatment in metastatic head and neck squamous cell carcinomas [110,111,112], another disease type commonly associated with oesophageal cancer. Hence, understanding why oesophageal cancers resist anti-EGFR therapies in spite of frequent EGFR amplifications will be important to design novel therapies targeting this pathway.

### 4.2. Targeting the VEGF Pathway in Oesophagel Cancers

Alterations in the vascular endothelial growth factor (VEGF) pathway are also common in oesophageal cancers, with 9–14% of these tumors showing *VEGFA* amplification, 5–6% having alterations in the Fms-Related Receptor Tyrosine Kinase 1 (*FLT1*) gene encoding for the VEGF receptor 1, and 3–4% alterations in the VEGFR receptor 2–encoding gene Kinase Insert Domain Receptor (*KDR*) [113]. VEGF has been targeted using sunitinib, a VEGFR 1-3 and platelet-derived growth factor receptor (PDGFR) targeting multikinase inhibitor, with or without concomitant chemotherapy. Neither sunitinib monotherapy nor the sunitinib-paclitaxel combination were superior to historical controls, with sunitinib plus chemotherapy also leading to increased toxicity [114,115]. Other multikinase inhibitors whose targets include VEGFR that have been tested in oesophageal cancer are sorafenib and anlotinib. Sorafenib mostly led to disease stabilization and it did not show promising results in terms of PFS [116]. Anlotinib was tested versus placebo in a randomized phase II study enrolling ESCC patients across 13 centers in China [116]. The study demonstrated a significant increase in the primary endpoint PFS (HR 0.46 (95%CI 0.32–0.66); *p* < 0.001), but no difference in terms of OS (HR 1.18 [95% CI 0.79–1.75]; *p* = 0.426). Aflibercept is a VEGF-A, VEGF-B, and PGF targeting fusion protein commonly used in metastatic colorectal cancer in combination with chemotherapy [117]. In a randomized phase II trial, patients with oesophageal, GEJ, or gastric adenocarcinoma were randomized to receive aflibercept or placebo in combination with FOLFOX [118]. Aflibercept was not effective in prolonging PFS (HR 1.11 [95%CI 0.64–1.91]; *p* = 0.72) or OS (HR 1.24 [95%CI 0.71–2.15]; *p* = 0.45) in this study population. Another randomized phase II clinical trial tested the addition of anti-VEGFR2 monoclonal antibody ramucirumab to FOLFOX chemotherapy in a cohort of previously untreated oesophageal, GEJ, and metastatic gastric cancer patients [119]. Of the 168 randomized patients, 48% were affected by oesophageal cancer. No benefit in terms of PFS (HR 0.98 [95%CI 0.69–1.37]; *p* = 0.886) or OS (HR 1.08 [95%CI 0.73–1.58]; *p* = 0.712) was apparent in the experimental arm of the ITT population. The oesophageal cancer subpopulation did not benefit either, neither in PFS (HR 1.30 (95%CI 0.81–2.09)) nor in OS (HR 1.10 [95%CI 0.61–1.97]). A further molecule targeting VEGFR2, apatinib, has been tested in a single-arm phase II study enrolling chemorefractory EAC, ESCC, and GEJ cancer patients in a single center in China [120]. In the 26 patients included in the efficacy analysis, the overall response rate (ORR) was 7.7% and the disease control rate was 61.5%, with a tolerable safety profile. Apatinib monotherapy was also recently tested in 40 chemorefractory ESCC patients enrolled in a phase II study in China [121]. In this cohort, treatment with apatinib led to an ORR of 7.5% and a DCR of 65.0%, but it was burdened by significant toxicity. Two of the treated patients had massive bronchopulmonary bleeding, and two further patients with unresected primary tumor experienced an oesophageal fistula. The current development of this small molecule is moving towards the combination with anti-PD1 treatment camrelizumab. In a recent single-arm phase II study conducted in Chinese patients that progressed after a first-line treatment, the combination led to impressive results, with a 40% ORR, including two complete responses [122]. Similarly impressive were the results from a phase II study enrolling first-line patients to receive apatinib, camrelizumab, liposomal paclitaxel and nedaplatin, followed by maintenance treatment with apatinib and camrelizumab [123]. A phase III trial randomizing ESCC patients to camrelizumab plus apatinib versus camrelizumab as second line treatment is yet to start recruiting [124]. While the results of this phase III trial will be eagerly awaited, the current landscape of treatments targeting the VEGF pathway in oesophageal cancer remains dismal, and much still needs to be done to implement targeted treatments in EAC and ESCC.

### 4.3. Targeting Other Pathways in Oesophageal Cancers

More pathways have been tested as therapeutic targets in oesophageal cancers, other than EGFR and VEGF. Claudins are transmembrane proteins involved in the structure of tight junctions, the main components of the epithelial–endothelial cell interaction [125]. Alterations in claudins expression have an association with carcinogenesis, which is consistent with the dysregulation of tight junctions that is thought to happen during tumor development [126]. Inside the claudin family, claudin 18 (CLDN 18) is highly expressed in healthy and tumor gastric tissue, particularly the 18.2 isoform (CLDN 18.2) [127]. A novel antibody targeting CLDN 18.2, zolbetuximab, has been tested in a phase II trial enrolling patients with oesophageal, GEJ, and gastric adenocarcinoma expressing CLDN 18.2 [128]. Zolbetixumab showed some antitumor activity, with 9% ORR across different dosing cohorts. Although just one patient with EAC was enrolled in the study, clinical trials testing combinations including zolbetixumab are ongoing in oesophageal cancers [129]. Cyclin-dependent kinases (CDK) 4 and 6 participate in the regulation of the cell cycle, and CDK4/6 inhibitors are currently used in combination with hormonotherapy in hormone receptor-positive breast cancer [130,131]. In ESCC patient-derived models, the loss of CDKN2A or CDKN2B predicts sensitivity to CDK4/6 inhibitors [132]. In oesophageal cancer patients, the CDK4/6 inhibitor palbociclib failed to demonstrate any significant clinical activity, leading to no objective responses in a small phase II clinical trial [133]. The hepatocyte growth factor (HGF)/mesenchymal–epithelial transition (MET) pathway has also been thoroughly studied in oesophageal and gastric cancer, due to the high frequency of MET overexpression in gastric cancer and to the association of MET dysregulation with tumor growth and metastatic spread [134,135]. The c-Met inhibitor tivantinib coupled with FOLFOX chemotherapy showed no benefit over historical controls in a phase II trial enrolling patients with previously untreated oesophageal, GEJ, or gastric adenocarcinoma [136]. Similarly, rilotumumab, an agent targeting the MET receptor HGF, did not lead to promising clinical benefit when added to FOLFOX [137].

Other frequent mutations, such as loss of the SWI/SNF ATPase subunit *ARID1A*, which is associated to a poor prognosis in patients with EAC, may benefit from targeted treatment [138]. Indeed, the loss of *ARID1A* modifies DNA damage response and thus may lead to a higher sensitivity to PARP inhibitors, as suggested by experiments on breast cancer epithelial cells in vitro [139]. In the same cell line, loss of *ARID1A* is associated to a higher sensitivity to PI3K and AKT inhibitors [140]. However, further studies will be required to determine whether EAC with alteration of *ARID1A* may benefit from targeted therapies.

Further molecular alterations that might be effectively targeted in oesophageal cancers include the fibroblast growth factor 2 (FGF-2). The fibroblast growth factor/fibroblast growth factor receptor (FGF/FGFR) signaling pathway plays a relevant role in the control of several cellular processes, including development, metabolism, and cell survival, and in the regulation of cancer stem cells [141,142]. By binding to FGFR1-4, FGF-2 mediates downstream signaling through, among others, the MAPK/ERK and the PI3K-AKT-mTOR pathways [142]. FGF-2 overexpression is associated with an increased risk of recurrence and worse overall survival in oesophageal cancer [143]. Although the pharmacological inhibition of the FGF/FGFR pathway with the FGFR1-3 inhibitor AZD4547 has shown promising activity in preclinical models [142], to our knowledge no clinical data are available so far. In addition, among 6667 tissue specimens from patients with advanced gastro-esophageal adenocarcinoma, only 269 (4.0%) FGFR2-altered cases were found [144]. This path may be, therefore, effectively targeted only in a limited proportion of patients.

Other molecular targets that have been evaluated in phase II clinical trials enrolling oesophageal cancer patients since 2010 include angiopoietins, heat shock protein 90 (HSP 90), and mTOR [145,146,147].

### 4.4. Basket and Tumor Agnostic Trials Relevant to Oesophageal Cancers

One targeted treatment that is currently being tested in several tumor types is sacituzumab govitecan (IMMU-132). IMMU-132 belongs to the antibody-drug conjugate class, treatments combining a monoclonal antibody targeting cancer-associated antigens with a cytotoxic drug through a chemical linker [148]. In IMMU-132, the antibody is directed against trophoblast cell surface antigen-2 (Trop-2), a calcium signal transducer implicated in embryonic development and in several oncogenic pathways that is upregulated in many epithelial tumor types [149]. The IMMU-132-01 phase I/II basket trial tested IMMU-132 in 495 subjects across several tumor types who had received at least one prior treatment [150]. In the 19 (3.8%) enrolled patients with oesophageal cancer (EAC or ESCC), IMMU-132 led to an ORR of 5.3%, with one partial response and no complete responses. The limited efficacy of IMMU-132 in gastrointestinal cancer types has been explained with previous exposure to topoisomerase I inhibitors such as irinotecan, a hypothesis that we could not verify in the IMMU-132-01 trial since data on prior treatments were not published.

While the drug development process in oncology has traditionally been tumor type specific, in recent years some tumor-agnostic (i.e., independent of histological tumor type) treatments have started appearing. The most frequent tumor-agnostic biomarker is microsatellite instability/mismatch repair deficiency (MSI/dMMR), which is effectively used to select patients for their sensitivity to anti-PD1/PD-L1 immune checkpoint inhibitors [151]. The prevalence of this alteration is dramatically different between EAC and ESCC and, as reported above, MSI/dMMR status is not the only biomarker predicting the efficacy of immune checkpoint inhibitors in oesophageal cancers [152]. Regarding targeted therapies, impressive results have been obtained with tumor-agnostic treatments targeting neurotropic tropomyosin receptor kinase (NTRK) fusions. The tropomyosin receptor kinase (TRK) signaling pathway, initially studied for its involvement in neuronal development, is increasingly being recognized as playing a significant role in carcinogenesis, and the most common way of TRK pathway oncogenic activation is fusions involving the NTRK1, NTRK2, or NTRK3 genes [153]. Although the prevalence of NTRK fusions is low among common solid tumors (<1%), the small population bearing these alterations derive great benefit from the two FDA-approved inhibitors larotrectinib and entrectinib (ORR across tumor types 79% and 59%, respectively) [154,155]. In oesophageal cancers, the prevalence of NTRK fusions has been reported to be 0.24%, making NTRK fusion an unviable treatment option for the vast majority of patients [156].

**Figure 2:**
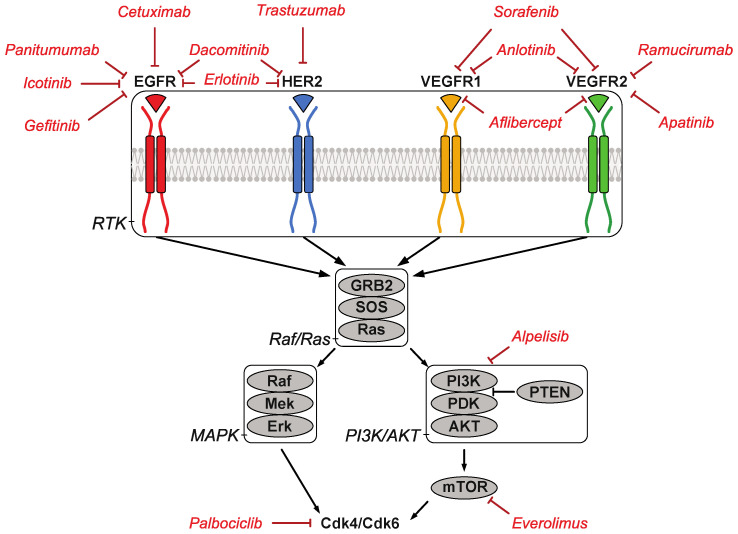
Scheme summarizing the signaling pathways that have been targeted the most frequently in oesophageal cancers. The different targeted therapies used in clinical trials to treat esophageal cancers are shown in red.

At this stage, the EGF and VEGF pathways have been the most frequent therapeutic targets tested in oesophageal cancers, with limited impact on survival except for treatments targeting HER2 (Figure 2). Clinical experience has shown that only a percentage of patients respond to therapies targeting Receptor Tyrosine Kinases (RTK), such as EGFRs and VEGFRs, even if their tumor expresses the altered target. This primary resistance to treatment is often due to constitutive activation of downstream signal transducers [157,158]. In addition, cancer cells may lose their ability to respond to the targeting RTK by activating alternative pathways (acquired resistance). It is therefore critical to investigate the role of downstream signal transducers such as the PI3K/AKT pathway.

## 5. Future Directions: Focus on the PI3K-AKT Pathway in Oesophageal Cancer

In spite of the frequency of the molecular alterations of PI3K in oesophageal cancer, the PI3K-AKT-mTOR pathway has received less attention than EGFR or VEGF in ESCC and EAC. Alterations of the PI3K pathway are frequent in oesophageal cancer: 10% of tumors present with *PIK3CA* mutations, 23% with *PIK3CA* amplifications, 10% with *PIK3CB* amplifications, and 3% with *AKT* mutations [159]. It is also noteworthy that the phosphorylation of the Akt protein, one of the mechanisms leading to the activation of the pathway, shows decidedly higher expression in ESCC than in corresponding normal tissue (90.4% versus 27.7%) [160], supporting the relevance of this path in tumor development. The PI3K-AKT-mTOR pathway controls processes involved in cell growth, metabolism, proliferation, and survival, and it is among the most frequently dysregulated signaling pathways in human cancers [159,161,162]. The pathway is normally activated by a multistep process starting from extracellular signals mediated by receptor tyrosine kinases (RTKs) and G-protein-coupled receptors (GPCRs) [163]. The catalytic subunit p110α of PI3Kα transduces these signals through the phosphorylation of the inositol ring of phosphatidylinositol-4,5-bisphosphate (PI-4,5-P2 or PIP2) to produce phosphatidylinositol-3,4,5-trisphosphate (PI-3,4,5-P3 or PIP3) [164]. The accumulation of PIP3 recruits Akt to the plasma membrane, and this translocation allows for the phosphorylation of the serine residue Ser473 of Akt by TORC2 [165]. The subsequent conformational change in Akt allows for the phosphorylation of its regulatory site Thr308 by the phosphoinositide-dependent kinase 1 (PDK1) [166]. Thus activated, Akt phosphorylates several proteins involved in cell survival, proliferation, and migration [167].

The PI3K-AKT pathway is antagonized by phosphatase and tensin homolog (PTEN), a lipid phosphatase converting PIP3 to PIP2 to inhibit the oncogenic AKT downstream signaling [168]. The tumor-suppressing role of PTEN is well known, and mutations in this gene frequently occur in solid tumors. For instance, approximately 10% of oesophageal carcinoma show mutations, amplifications, deletions, or multiple alterations of PTEN [113]. Perhaps less characterized is the role of inositol polyphosphate 5-phosphatases in regulating the PI3K-AKT-mTOR pathway. Inositol polyphosphate 5-phosphatases (IP5P) are a family of ten signal-modulating enzymes regulating a number of cellular functions through the regulation of phosphoinositides such as PIP2 and PIP3 [169,170]. Alterations in the IP5P family members have been shown to have a context-dependent oncogenic or tumor suppressor role in several solid tumors [171,172,173,174], and the pharmacological targeting of the IP5Ps SHIP1 and SHIP2 has shown promise in preclinical models of breast cancer [175].

Even though the pharmacological inhibition of mTOR in EAC patients has led to limited clinical benefit so far [146], evidence exists regarding the potential anti-tumor effect of targeting the PI3K-AKT-mTOR pathway in ESCC preclinical models, through direct inhibition or via the suppression of upstream targets. AKT itself is a potential target in ESCC, as shown by the effect of AKT pharmacological inhibition in cell lines and in vivo [176,177]. Specific inhibition of PI3Kα can be achieved with the use of pharmacological inhibitors such as alpelisib, which is currently marketed for use in metastatic breast cancer [178,179]. PI3Kα inhibitors inhibit proliferation in ESCC cell lines, but resistance inevitably ensues through multiple mechanisms, including the hyperactivation of mTORC1, the mitogen-activated protein kinase (MAPK) pathway, and c-Myc [180]. The concomitant treatment with PI3Kα and MEK/mTORC1/BET inhibitors seems effective in restoring sensitivity and suppressing tumor cell growth in resistant models. Alpelisib is currently being tested in a phase Ib/II trial in combination with the anti-HER3 inhibitor LJM716 in patients with previously treated ESCC [181]. It will be important to assess the impact of inhibitors of the PI3K-AKT-mTOR pathway in the near future to determine to what extent this pathway could be more efficient than the EGFR pathway for oesophageal cancer growth and progression. Hopefully, targeting transducers that are common to several pathways may be more efficient than targeting receptors, whose activity can be somehow easily compensated in cancer cells. Drug combinations and association with immunotherapy may be the most promising strategies to circumvent potential resistance mechanisms.

## 6. Conclusions

The discovery of effective treatments for oesophageal cancer is an unmet need in oncology, as reflected by the dismal prognosis of patients with unresectable and metastatic disease [1]. After years with limited to no changes in terms of novel therapies for metastatic oesophageal cancer, the recent introduction of immune checkpoint inhibitors has revolutionized the therapeutic landscape and will significantly impact the life expectancy of these patients [34], while the development of targeted treatments is still lagging behind. Although the design of therapies aiming at specific alterations is expected to benefit from the ongoing effort to perfect the molecular characterization of oesophageal cancer, the emerging complexity of this disease is posing novel challenges to overcome. First of all, oesophageal cancer cannot be considered a single entity, but rather a collection of diseases differing from the histological (ESCC versus EAC) and molecular point of view [44,182,183]. While most clinical trials currently enroll all oesophageal cancer patients, intertumoral heterogeneity in oesophageal cancer will need to be taken into account to achieve effective drug development. Second, oesophageal cancer heterogeneity is evident not only at the inter- but also at the intratumoral level [66]. Intratumoral heterogeneity limits the effectiveness of treatments targeting a single molecular alteration, due to the presence of tumoral clones that depend on alternative pathways of development [184]. Combining treatments represents a potential method to circumvent this issue while reducing toxicity [185]. Deeper characterization of esophageal cancer by using single-cell omics and spatial transcriptomics will certainly provide important data about the intra-tumoral heterogeneity, the biology of the sub-clones, their interactions, as well as the role of the microenvironment in tumor biology [183,186]. For instance, single-cell RNA sequencing recently showed high expression of Lymphocyte Activating 3 (LAG3) in NKT/CD8+ T-cells in ESCC samples, supporting a potential role for anti-LAG3 treatments (a novel class of immune checkpoint inhibitors) in this tumor type [186]. This level of detail will be crucial to design efficient targeted therapies in the future.

The deepening of our understanding of the molecular mechanisms underpinning the development of EAC and ESCC is offering novel insights into new targets for molecularly selected treatments. Renewed efforts aiming at exploiting the molecular mechanisms of cancer growth will bring about significant changes in the therapeutic landscape of patients with oesophageal cancers, with the hope of dramatically increasing the life expectancy of this population.

## Figures and Tables

**Table 1:** Immune checkpoint inhibitor trials in metastatic oesophageal cancer.

Study	*N*	Tumor Type(s)	Phase	Treatment Line	Control Arm	Experimental Arm	Molecular Selection	Primary Endpoint	mPFS	mOS
KEYNOTE-590	749 (OC 658)	OC, GEJ	III	First line	CF + Placebo	CF + Pembrolizumab	No	OS	6.3 vs. 5.8 HR 0.65 *p* < 0.0001	12.4 vs. 9.8 HR 0.73 *p* < 0.0001
CheckMate-649	955 ^§^	OC, GEJ, GC	III	First line	CAPOX or FOLFOX	CAPOX or FOLFOX + NIVOLUMAB	CPS ≥ 5	OS/PFS	7.7 vs. 6.0 HR 0.68 *p* < 0.0001	14.4 vs. 11.1 HR 0.71 *p* < 0.0001
CheckMate-648	315 °	ESCC	III	First line	CF	CF + Nivolumab	PD-L1 ≥ 1%°	OS/PFS	6.9 vs. 4.4 HR 0.65 *p* = 0.0023	15.4 vs. 9.1 HR 0.54 *p* < 0.0001
KEYNOTE-181	628 *	OC	III	Second line	Paclitaxel, docetaxel, or irinotecan	Pembrolizumab	No *	OS	2.1 vs. 3.4 HR 1.11 *p* not reported	7.1 vs. 7.1 HR 0.89 *p* = 0.056
Attraction-3	419	ESCC	III	Second line	Paclitaxel or docetaxel	Nivolumab	No	OS	1.7 vs. 3.4 HR 1.08 *p* not reported	10.9 vs. 8.4 HR 0.77 *p* = 0.019

Abbreviations—CF: cisplatin + 5-fluorouracil; CPS: combined positive score; ESCC: oesophageal squamous cell carcinoma; GEJ: gastro-oesophageal junction; GC: gastric cancer; HR: hazard ratio; (m)OS: (median) overall survival; (m)PFS: (median) progression-free survival; OC: oesophageal cancer; PD-L1: programmed death ligand 1. ^§^ Primary endpoints were OS and PFS in the CPS ≥ 5 population. OS and PFS results for all randomized patients (*N* = 1581) and for the immunotherapy-only arm are not reported here. ° Primary endpoints were OS and PFS in the PD-L1 ≥ 1% population. OS and PFS results for all randomized patients (*N* = 970) and for the immunotherapy-only arm are not reported here. * Primary endpoints were OS in the CPS ≥ 10 population, in the ESCC population, and in all patients. Reported here are the results in all randomized patients.

**Table 2:** Targeted therapies trials in metastatic oesophageal cancer.

Study (Publication Year)	*N*	Tumor Type(s)	Phase	Treatment Line	Control Arm	Experimental Arm	Molecular Target	Primary Endpoint	mPFS (Months)	mOS (Months)
EGFR-targeting agents										
REAL3 (2013)	553 (OC 217)	OC, GEJ, GC	III	First line	EOC	mEOC + Panitumumab	EGFR	OS	6.0 vs. 7.4 HR 1.22 *p* = 0.07	11.3 vs. 8.8 HR 1.37 *p* = 0.01
COG (2014)	449 (OC 352)	OC, GEJ	III	Second, third, later lines	Placebo	Gefitinib	EGFR	OS	1.6 vs. 1.2 HR 0.80 *p* = 0.02	3.7 vs. 3.7 HR 0.90 *p* = 0.29
POWER (2020)	146 (OC 146)	ESCC	III	First line	CF	CF + Panitumumab	EGFR	OS	5.3 vs. 5.8 HR 1.21 *p* = 0.29	9.4 vs. 10.2 HR 1.17 *p* = 0.43
TRIO-013/LOGIC (2016)	487 (OC 20)	EAC, GEJ, GC	III	First line	CAPOX + Placebo	CAPOX + Lapatinib	HER2	OS	6.0 vs. 5.4 HR 0.82 *p* = 0.038	12.2 vs. 10.5 HR 0.91 *p* = 0.35
AGITG ATTAX3 (2016)	77 (OC 28)	OC, GEJ, GC	II	First line	DCF	DCF + Panitumumab	EGFR	ORR	6.0 vs. 6.9 HR NR p NR	10.0 vs. 11.7 HR NR p NR
Janjigian Y et al. (2020)	37 (OC 14)	EAC, GEJ, GC	II	First line	/	Trastuzumab + Pembrolizumab + Capecitabine + Oxaliplatin/Cisplatin	HER2	PFS	13.0	27.3
Yoon H et al. (2018)	18	EAC	II	Second line	/	Irinotecan + Panitumumab	EGFR	ORR	2.9	7.2
Wainberg ZA et al. (2011)	38 (OC 12)	EAC, GEJ	II	First line	/	FOLFOX + Erlotinib	EGFR	ORR	5.5	11.0
Hong MH et al. (2020)	49	ESCC	II	Second, third, later lines	/	Afatinib	EGFR, HER2, HER4	ORR	3.4	6.3
Huang J et al. (2016)	54	ESCC	II	Second, third, later lines	/	Icotinib	EGFR	ORR	1.7	5.0
Kim HS et al. (2015)	49	ESCC	II	Second, third line	/	Dacomitinib	HER1, HER2, HER4	ORR	3.3	6.4
Ilson DH et al. (2011)	30	OC, GEJ	II	First, second line	/	Erlotinib	EGFR	ORR	NR	10.3
Chan JA et al. (2011)	35 (OC 12)	EAC, GEJ, GC	II	Second, third line	/	Cetuximab	EGFR	ORR	1.6	3.1
SWOG 0415 (2010)	55	EAC, GEJ	II	Second line	/	Cetuximab	EGFR	OS	1.8	4.0
Angiogenesis-targeting agents										
ZAMEGA (2019)	64 (EAC 21)	EAC, GEJ, GC	II	First line	FOLFOX + Placebo	FOLFOX + Aflibercept	VEGF-A, VEGF-B, PGF	PFS	9.7 vs. 7.4 HR 1.11 *p* = 0.72	14.5 vs. 18.8 HR 1.24 *p* = 0.45
Yoon HH et al. (2016)	168 (OC 80)	OC, GEJ, GC	II	First line	FOLFOX + Placebo	FOLFOX + Ramucirumab	VEGFR2	PFS	6.4 vs. 6.7 HR 0.98 *p* = 0.89	11.7 vs. 11.5 HR 1.08 *p* = 0.71
Yanwei L et al. (2020)	26 (OC 26)	OC	II	Second, third, later lines	/	Apatinib	VEGFR2	RR	4.6	6.6
ESO-Shanghai 11 (2021)	40 (OC 40)	ESCC	II	Second, third, later lines	/	Apatinib	VEGFR2	PFS	3.8	5.8
Zhang B et al. (2020)	30	ESCC	II	First line	/	Camrelizumab + Liposomal irinotecan + Nedaplatin + Apatinib	VEGFR2	ORR	6.9	19.4
Wu C et al. (2015)	25 (OC 15)	OC, GEJ	II	First, second, third line	/	Sunitinib	VEGFR 1-3, PDGFR	PFS	1.6	3.9
Schmitt JM et al. (2012)	28 (OC 22)	OC, GEJ	II	First, second line	/	Paclitaxel + Sunitinib	VEGFR 1-3, PDGFR	ORR	3.7	7.5
Huang J et al. (2021)	165	ESCC	II	Second, third, later lines	Placebo	Anlotinib	VEGFR 1-3, FGFR 1-4, PDGFR α,β, Ret, c-Kit	PFS	3.0 vs. 1.4 HR 0.46 *p* < 0.001	6.1 vs. 7.2 HR 1.18 *p* = 0.426
Janjigian Y et al. (2015)	34 (OC 23)	ESCC, GEJ	II	Second, third line	/	Sorafenib	VEGFR2, PDGFR, RET, RAF1	PFS	3.6	9.7
Other molecular targets in oesophageal cancers										
MONO (2019)	54 (OC 1)	EAC, GEJ, GC	II	Second, third, later lines	/	Zolbetuximab	CLDN 18.2	ORR	NR	NR
Karasic et al. (2020)	21 (OC 13)	OC, GEJ, GC	II	Second, third, later lines	/	Palbociclib	CDK4/6	ORR	1.8	3.0
PRODIGE-17 (2019)	57 (OC 8)	EAC, GEJ, GC	II	First line	/	FOLFOX + Rilotumumab	HGF	PFS	7.6	11.5
Pant S et al. (2017)	34 (OC 14)	EAC, GEJ, GC	II	First line	/	FOLFOX + Tivantinib	C-MET	ORR	6.1	9.6
Goyal L et al. (2020)	26 (OC 7)	OC, GEJ, GC	II	Second, third line	/	Ganetespib	HSP 90	ORR	2.0	3.1
Wainberg ZA et al. (2015)	45 (OC 11)	EAC, GEJ, GC	II	Second, third line	/	Everolimus	mTOR	DCR	1.8	3.4
Eatock MM et al. (2013)	171 (OC 30)	EAC, GEJ, GC	II	First line	Cisplatin + Capecitabine + Placebo	Cisplatin + Capecitabine + Trebananib	Angiopietins 1/2	PFS	4.2 vs. 5.2 HR 0.99 *p* = 0.96	9.1 vs. 12.8 NR NR

Abbreviations—CDK: cyclin-dependent kinase; CF: cisplatin + 5-fluorouracil; CLDN 18.2: claudin 18.2; DCF: docetaxel + cisplatin + fluorouracil; EAC: oesophageal adenocarcinoma; EGFR: epidermal growth factor receptor; EOC: epirubicin + oxaliplatin + capecitabine; ESCC: oesophageal squamous cell carcinoma; GEJ: gastro-oesophageal junction; GC: gastric cancer; HER1-4: human epidermal growth factor receptor 1–4; HGF: hepatocyte growth factor; HR: hazard ratio; HSP: heat shock protein; (m)OS: (median) overall survival; (m)PFS: (median) progression-free survival; mTOR: mammalian target of rapamycin; NR: not reported; PDGFR: platelet-derived growth factor; PGF: placental growth factor; RT: radiation therapy; VEGF: vascular endothelial growth factor; VEGFR1-3: vascular endothelial growth factor receptor 1–3.
